# The virtual hallway consult as an effective means of continuing professional development in physiatry

**Published:** 2017-12-15

**Authors:** Karen Ethans, Tim Deutscher, Mayur Nankar

**Affiliations:** 1 University of Manitoba, Manitoba, Canada; 2 Health Science Centre, Manitoba, Canada; 3 Island Health, Nanaimo, British Columbia, Canada; 4 University of British Columbia, British Columbia, Canada

## Abstract

**Background:**

A personal learning project (PLP) is an accredited form of Continuing Professional Development (CPD) in Canada, and is a self-initiated learning activity that is stimulated by a question, issue or dilemma in one’s professional practice. Many complex cases or issues have no answers that are readily available. Many physicians rely day to day on other physician colleagues that they may consult in their institution. Given the paucity of same specialty Physical Medicine and Rehabilitation colleagues in Canadian centres, the idea of Virtual Spinal Cord Injury (SCI) Hallway germinated, to provide a simple tool to extend our hallways to reach colleagues with similar interests across the country.

**Methods:**

The Virtual SCI Hallway is a service set up on Yahoo Groups, with no cost to the users. On this invite-only site, members may post a question, and then all members receive the post by email. Any member may respond.

**Results:**

The SCI Hallway has been running successfully for over 13 years. As of January 2017, there were 38 members, with 2124 messages within approximately 324 conversations. Activity has been consistent since 2003. Questions and posts are not always medical expert related; there are also advocacy, professional, and scholar-role related posts.

**Discussion:**

Communication amongst specialists about practice and management of complicated problems is important for CPD, yet is difficult in subspecialized areas of medicine. Although there are many chat-pages in different areas of medicine on the internet, to our knowledge, there is not another secure, invite-only site that is low-maintenance and no cost.

## Introduction

Continuing medical education (CME) is vital for practicing physicians to ensure that they can incorporate new ideas into practice and to affirm that previous knowledge and practice regimes are still up to date. CME is one form of Continuing Professional Development (CPD), which is an all-encompassing term that Canada’s Royal College of Physicians and Surgeons of Canada (RCPSC) has used to reflect the learning needs of the many CanMEDS^1^ roles of physicians, not just the medical expert role. Given the importance of all the roles in medicine, CPD is the term used by the RCPSC and will be referred to henceforth in this document.

A personal learning project (PLP) is an accredited form of CPD in Canada, and is a self-initiated learning activity that is stimulated by a question, issue or dilemma in one’s professional practice.^2^ The process of carrying out a PLP, as suggested on the RCPSC website, is as follows: Step 1: Identify your question, Step 2: Develop a plan to search for evidence, Step 3: Come to conclusions, Step 4: Document your project in MAINPORT ePortfolio. Included in this documentation is the question pursued, the time spent learning, the day the project was completed, and the outcomes or conclusions that the learner reached. PLP’s are powerful forms of CPD, as they are created by the physician to identify and solve questions of their own learning needs, in contrast to the typical attendance at rounds that may or may not have a specific learning need for that individual physician. Given that PLP’s are likely one of the most effective means of CPD, the physician is instructed to claim two credits per hour for this type of learning, with no limit to the number of credits per year. Using the ePortfolio within MAINPORT not only allows the user to track their CPD points, but gives the opportunity for the user to reflect on the learning done, and how this learning may be further pursued, as well as how to use the learning to incorporate making changes in practice. 3

Being able to find and research a helpful answer to a physician’s learning need can be challenging. Many complex cases or issues have no answers that are available by doing simple literature searches or reading textbooks or clinical practice guidelines. Many physicians rely day to day on other physician colleagues that they may consult formally, or informally, in their institution. However, given that Canada is a geographically vast and not densely populated country, in many cases there are few or no physician colleagues to consult within their office or institution, physicians that have the experience or expertise that is needed to help the physician solve the problem that they are facing.

Specialists in Physical Medicine and Rehabilitation (PM&R) in particular find this challenging; in many areas there may only be one physiatrist in the city or even in that part of the province. For example, Newfoundland has one physiatrist in the western part of the province, and one in the eastern part. Even within the academic centres, there is often only one physiatrist with a subspecialty expertise for specific patient populations such as spinal cord injury (SCI) or traumatic brain injury. People with SCI have numerous systems affected, and often present with very complex issues, for which answers cannot be found with literature searches or textbooks, leaving the attending physiatrist wishing to consult with colleagues for advice. In medicine, there has been a long tradition of having “hallway consults” to obtain practical and real world timely and useful information directly from our colleagues. Given the paucity of same specialty PM&R colleagues in Canadian centres, the idea of Virtual Rehabilitation Hallways (“Hallways”) germinated. The idea is to provide a simple tool to extend our hallways to reach colleagues with similar interests across the country. Thus, the purpose of this paper is to report on the use of such an educational tool for CPD, in particular to be used as a key and practical source for PLPs in the area of SCI medicine, in which we have been using a specific Hallway for SCI issues (SCI Hallways).

## Methods

The Hallways is a service set up on Yahoo Groups, which makes some revenue from advertisements, thus there is no cost to the users. Our group developed several Hallways that cover many areas of Physical Medicine and Rehabilitation, including SCI, Stroke, Brain Injury, Pain (including musculoskeletal rehab and other pain issues), Prosthetics and Orthotics, Pediatric Rehabilitation. Many of these have not been very active since inception in 2001; however, the SCI-Hallway has been running successfully for over 13 years.

The focus of this paper is to report on the Hallway developed for SCI specific issues, theSCI Hallway. To join the SCI Hallway, a physician needs to be invited by the moderator. The moderator can be emailed directly to receive this invite. Once a member of the SCI-Hallway the physician can post regarding a particular question related to individual patient management or related to management in of a specific area in that patient population. The question is posted either on the Yahoo website, or through email. Once the question is posted, each physician member registered on the group receives the email, which comes from the SCI Hallway site rather than the physician’s personal email. Any member of the group may respond. The responses may be personal professional experience and expertise, links to other information, or papers on the subject matter. As this report does not involve research on human subjects, but rather reports concept and positive use of this education system, with examples of ideas shared, this study was exempt from ethics review.

To ensure that personal health information is never revealed, any patient-identifying information is not allowed, and the moderator checks all content to ensure that this guideline is followed; any posts that may potentially have identifying information are immediately removed and the physician responsible for the post is reminded of the guidelines.

If colleagues respond to the post with information or advice that the physician finds useful, and /or posts link to websites or papers that help guide the physician in patient management, the physician then can follow up by reviewing the information provided, gather any additional information that is needed to better answer the question, and often may provide follow up information for his/her colleagues via the SCI-Hallway. The physician may then add this to their CPD profile as a PLP by entering the question, the resolution and how it might change practice/behavior, and then enter the number of hours spent on the project, claiming twice that as credit hours.

To assess use of the SCI Hallway, we reviewed the records of use since inception in 2001. All of the conversations remain on the Yahoo website and can only be accessed by the members of the group. The system automatically keeps conversations on a subject line together as one conversation, with all responses bundled within the conversation. Subjects were reviewed by the authors to ensure that topics were appropriate (i.e., related to SCI medicine) and that only one subject was discussed per conversation. If there was a conversation without a subject line, the topic of conversation did not get bundled and this had to occur manually.

## Results

**Figure 1:**
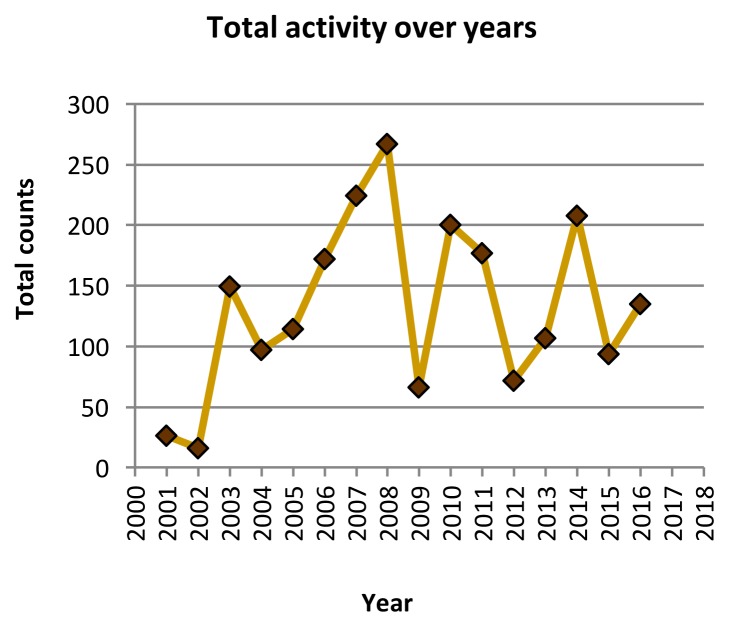
Consistent activity of the SCI-Hallway since 2001

As of January 2017, there were 38 members on the SCI-Hallway. The members are distributed across Canada as follows: British Columbia 10, Alberta 6, Saskatchewan 1, Manitoba 1, Ontario 10, Quebec 5, New Brunswick 1, Nova Scotia 2, other 2 (both previously in Canada now in California (1) and Saudi Arabia (1). All members requested invites and none were declined. All members were active members of our national association and were known to the moderator; thus, all requests were accepted. No one have requested to leave the group. Since inception in 2001, there were 2,124 messages, within approximately 324 conversations. The use has been consistent since 2003; the first two years were slow (Figure 1).

In addition to medical expert questions there were conversations relating to other CanMEDS physician roles such as notices of grant or job opportunities, and interdisciplinary team structure advice. Some examples include advocate (a call for donations to an international disaster relief fund for SCI rehab efforts in another country), scholar (notification of grant application opportunities in SCI medicine), collaborator (questions about what allied health services are offered through the SCI rehab programs in Canada such as recreational therapy so as to advocate with local health authorities on such issues), and leader and professional (a call for applications of the head of the SCI program in their city). Subjects under medical expert that have appeared frequently include management of aggressive heterotopic ossification, difficult spasticity (including baclofen pump complications), autonomic dysreflexia, orthostatic hypotension, neuropathic pain, and pregnancy issues in females with SCI. A recent discussion was put forward with concerns arising about a patient who complained of episodes of autonomic dysreflexia during sexual activity. This is a life threatening condition of severe elevation of blood pressure. As with many people with SCI, the patient used sildenafil for obtaining and maintaining erections during sexual activity. As such, use of prophylactic antihypertensive medications pre-sexual activity were contraindicated while using sildenafil. Thus, the physician posted this scenario on the Hallway, and a few colleagues responded that they had had similar concerning scenarios in the past. One colleague, however, responded that they had some success prescribing low dose clonidine for this situation, as it is relatively effective as a prophylactic medication for preventing autonomic dysreflexia, without causing significant drop in blood pressure. The physician then met with the patient again, discussed the potential risks, and prescribed the clonidine with patient consent. The patient found that this worked well for him, and had no further severe episodes of autonomic dysreflexia during sexual activity.

The advertising, which allows this service to be free, has remained tasteful and minimally intrusive, with no complaints from users.

## Discussion

Communication amongst specialists and subspecialists about practice patterns, management of complicated problems, and up-to date literature is important for CPD, yet is difficult in areas of medicine that are very subspecialized. Previous reports have evaluated online support tools for providing formal consultation with success.^4^ However, the purpose of the Hallways is not for formal consultation, but rather to get trusted advice from fellow experts. Given that the membership must be invited, this measure ensures that members have adequate credentials and that unsolicited questions from the public do not appear. To date, there have been no breaches, i.e., no members of the public or any other non-members have been able to access or post on the SCI Hallway. While there are many chat pages serving different areas of medicine, and there are other secure options for similar exclusive use, our secure, by invitation only, low-maintenance, no-cost site appears to be unique.

PLPs remain an effective tool for CPD for individual physicians, and are likely especially useful for those who are remote geographically from other physicians with similar practice patterns. There are now different categories of PLPs on the Mainport system as follows:

- Address clinical or academic questions
- Preparation for formal teaching activities
- Development of research activities
- Address medical-professional administrative or systems related questions/issues
- Other

Obtaining expert advice from colleagues with similar practices could assist in all of these categories of PLP, and traditionally such gathering of advice from colleagues has long been an important and practical method of collecting answers to solving a question in the physician’s clinical or academic questions in particular. Using the ePortfolio to log the PLP may help physicians further evaluate how to incorporate what they have learned in the virtual hallway consult to reflect upon how to incorporate their learning into better practice.

### Conclusion

The SCI Hallway site has been consistently active since 2003 with physiatrists seeking CPD advice about SCI management. This is a practical way of having specialists seek answers to PLPs, especially with getting advice regarding real-life, complex patient issues. Usually occurring in the hallways of the institution, inter-physician consultation can now run across the country and potentially around the world.

## References

[1] CanMEDS: Better standards, better physicians, better care. [Internet].

[2] Royal College of Physicians and Surgeons of Canada (2015). Personal Learning Projects: A Four Step Guide. Dialogue.

[3] Gordon JA1, Campbell CM (2013). The role of ePortfolios in supporting continuing professional development in practice. Med Teach.

[4] Lacasta Tintorer D1, Flayeh Beneyto S, Alzaga Reig X, Mundet Tuduri X, De la Fuente JA, Manresa JM, Torán Monserrat P, Saigí Rubió F (2013). Impact of the implementation of an online network support tool among clinicians of primary health care and specialists: ECOPIH Project. BMC Fam Pract.

